# Learning curve comparison between switching approach and switching implant in cementless short stem total hip arthroplasty

**DOI:** 10.1007/s00402-024-05518-9

**Published:** 2024-09-09

**Authors:** Christian Stadler, Jonas Sebastian Bolm, Clemens Schopper, Bernhard Schauer, Matthias Holzbauer, Tobias Gotterbarm, Matthias Luger

**Affiliations:** 1 Johannes Kepler University Linz, Altenberger Strasse 96, Linz, 4040 Austria; 2 Department for Orthopaedics and Traumatology, Kepler University Hospital GmbH, Med Campus III, Krankenhausstraße 9, Linz, 4020 Austria

**Keywords:** Direct anterior approach, Cementless short stem, Learning curve, Total hip arthroplasty

## Abstract

**Introduction:**

Implementing new approaches or new implants is always related with a certain learning curve in total hip arthroplasty (THA). Currently, many surgeons are switching to minimally invasive approaches combined with short stems for performing THA. Therefore, we aimed to asses and compare the learning curve of switching from an anterolateral Watson Jones approach (ALA) to a direct anterior approach (DAA) with the learning curve of switching from a neck-resecting to a partially neck-sparing short stem in cementless THA.

**Materials and methods:**

The first 150 consecutive THA performed through a DAA (Group A) and the first 150 consecutive THA using a partially neck-sparing short stem (Group B) performed by a single surgeon were evaluated within this retrospective cohort study. All cases were screened for surgery related adverse events (AE). Furthermore, the operative time of each surgery was evaluated and the learning curve assessed performing a cumulative sum (CUSUM) analysis.

**Results:**

Overall, significantly more AE occurred in Group A compared to Group B (18.0% vs. 10.0%; *p* = 0.046). The sub-analysis of the AE revealed higher rates of periprosthetic joint infections (2.7% vs. 0.7%; *p =* 0.176), periprosthetic fractures (4.0% vs. 2.0%; *p* = 0.310) and overall revisions (4.7% vs. 1.3% *p =* 0.091) within Group A without statistical significance. The CUSUM analysis revealed a consistent reduction of operative time after 97 cases in Group A and 79 cases in Group B.

**Conclusion:**

A significantly higher overall rate of AE was detected while switching approach compared to switching implant for performing THA. However, according to the results of this study, surgeons should be aware of the learning curve of the adoption to a new implant with different fixation philosophy as well.

**Supplementary Information:**

The online version contains supplementary material available at 10.1007/s00402-024-05518-9.

## Introduction

Minimally invasive (MIS) techniques have been progressively evolving within the last decades across many surgical fields [1–4]. While in orthopedics, traditional approaches in total hip arthroplasty (THA) have proven reliability over many years, MIS approaches in combination with modern short curved stems are becoming increasingly popular [5–8]. Potential advantages of MIS THA include smaller skin incisions with less soft tissue damage and pain resulting in earlier mobilization and improved early patient related outcomes [9, 10]. However, switching to MIS approaches in THA is associated with a significant learning curve [11–14]. Especially switching to a direct anterior approach (DAA) to the hip is known to be a challenging task even for experienced orthopedic surgeons [15].

Compared to standard straight hip stems, modern short curved hip stems enable more sparing of soft tissue and bone stock with decreased risk of trochanteric fractures, while still enabling accurate reconstruction of the pre-arthritic hip biomechanics with more physiological load distribution within the proximal femur [16–22].

While the learning curve of switching to a DAA for THA is well investigated, less data is available regarding the learning curve of switching between short stems with different levels of femoral neck osteotomy (e.g. neck-resecting stems vs. partially neck-sparing stems) and consecutively different fixation philosophies [23, 24]. Therefore, we sought to compare two different learning curves: 1.the learning curve of implementing the DAA and  2. the learning curve of switching the type of short stem in DAA THA. The aim was to compare, if switching the approach or switching the type of short stem leads to a difference in complications during the learning curve of one single, fellowship trained, surgeon.

## Materials and methods

### Study population and medical record evaluation

The first learning curve consisted of the first consecutive 150 cases of one single, fellowship trained, surgeon, who was switching from an anterolateral MIS approach in supine positioning using a cementless short stem (Fitmore^®^, ZimmerBiomet, IN, Warsaw, USA) to DAA (Group A). In Group A the surgeon was familiar with the type of stem but was transitioning to the DAA. The second learning curve consisted of the first consecutive 150 cases of the same surgeon after switching from a neck-resecting short stem (Fitmore^®^, ZimmerBiomet, IN, Warsaw, USA) to a partially neck-sparing short stem (ANA.NOVA alpha proxy^®^, ImplanTec GmbH, Moedling, Austria; Group B). These cases were performed following the cases of Group A. In Group B the surgeon was therefore familiar with the approach (DAA) but was transitioning to a new type of short stem. Surgeries were performed between January 1st 2017 and March 31st 2023. Patients with primary osteoarthritis of the hip, osteoarthritis following mild hip dysplasia (Crowe I) or avascular necrosis of the femoral head undergoing unilateral or bilateral THA receiving one of the two implants mentioned above were included in this study. Patients with severe hip dysplasia (Crowe > I), posttraumatic osteoarthritis of the hip or other conditions affecting the distal epiphyseal or metaphyseal bone stock received other implants (straight stems) and were therefore excluded from this study. The medical record of each patient was screened retrospectively for basic demographic data. Furthermore, all patient files including the surgical protocol were evaluated regarding the operative time, length of stay at the hospital, intraoperative and postoperative adverse events (AE) within the first 90 days after surgery. AE were classified according to an adapted Clavien-Dindo classification [25]. Postoperative anemia was defined as hemoglobin levels below 8.0 g/dL with a need of blood transfusion. Prolonged wound secretion or delayed epidermal closure of more than 14 days postoperatively without signs of inflammation were documented as AE. According to the recommendation of the U.S. Food and Drug Administration (FDA) potentially life-threatening events and conditions as well as other events causing revision, permanent impairment or prolonged hospitalization were classified as “serious AE” [26].

This retrospective cohort study was approved by the local ethics committee (reference number: 1094/2023). The need of informed consent was waived by the ethics committee due to the retrospective study design. Alle procedures were performed in accordance with the principles of the Declaration of Helsinki.

### Surgical technique

A standardized perioperative treatment protocol was applied in all cases. All patients preoperatively received a single shot antibiotic prophylaxis (1,5 g of cefuroxime or 600 mg of clindamycin in case of penicillin allergy) and tranexamic acid (20 mg per kilogram of body weight). A MIS DAA in supine position was performed in all cases without the use of a traction table but with kinking the table for stem preparation and performance of a lazy figure of four with the affected leg placed beneath the contralateral leg [27]. The neck-resecting Fitmore^®^ hip stem (ZimmerBiomet, Warsaw, IN, USA) combined with the press-fit Allofit^®^ acetabular cup (ZimmerBiomet, Warsaw, IN, USA) was used within Group A, while the partially neck-sparing ANA.NOVA alpha proxy^®^ hip stem (ImplanTec GmbH, Moedling, Austria) in combination with the press-fit ANA.NOVA alpha^®^ acetabular cup (ImplanTec GmbH, Moedling, Austria) was used within Group B. According to the recommended level of the femoral neck osteotomy, the Fitmore^®^ hip stem (ZimmerBiomet, Warsaw, IN, USA) can be classified as a neck-harming short stem [28, 29]. It has a triple tapered design and is available in 14 different sizes with four different offset options for each size [30]. The ANA.NOVA alpha proxy^®^ stem (ImplanTec GmbH, Moedling, Austria) can be classified as partially neck-sparing short stem according to the recommended level of femoral neck osteotomy [28, 29]. It has a triple tapered design which aims for a calcar guided press fit 3-point anchorage with the medial calcar and the lateral cortex as main fixation zones. It is available in 12 different sizes with two different offset options for each size [31].

All components were implanted according to the instructions of the manufacturer. Full weight bearing was permitted immediately after THA.

### Statistical analysis

SPSS version 28 (IBM, Chicago, IL, USA) was used for performing the statistical analyses. For metric scaled data, arithmetic mean value and standard deviation were calculated. To test for normal distribution, Kolmogorov-Smirnov-Test was performed. Chi-Square-Test including Odds-Ratio (OR) calculation was performed for analyzing normally distributed categorial parameters and t-Test for analyzing normally distributed metric parameters. Mann-Whitney-U-Test was used for evaluating non-normally distributed metric parameters. Cumulative sum (CUSUM) analysis was performed as previously described in order to determine the learning curve regarding the operative time within both study groups [32]. The cases of each group were evaluated in chronological order. The CUSUM value of the first case of each group was the difference between the operative time of the first case and the average operative time of the study group. The CUSUM values of the following cases were calculated determining again the difference between the operative time of the particular case and the average operative time of the study group and additionally adding the CUSUM value of the previous case [32]. A *p* value < 0.05 was considered as statistically significant.

## Results

A total of 300 THAs were included in this study (Group A: *n* = 150; Group B: *n* = 150), THA was performed bilaterally in 7 patients (Group A: *n* = 4; Group B: *n* = 3). The mean age of the study population was 66.6 ± 10.9 years with a mean BMI of 27.5 ± 4.6 and a mean ASA-Score of 2.0 ± 0.6 with no significant difference between the two study groups (Table 1).

**Table 1:** Demographics of the study population

	Group A (*n* = 150)	Group B (*n* = 150)	Overall (*n* = 300)	*p* Value
Age (y)	66,1 ± 10,9	67,0 ± 10.9	66,6 ± 10,9	0,499
Height (cm)	169,7 ± 8,8	170,9 ± 9,7	170,3 ± 9,3	0,288
Weight (kg)	80,3 ± 16,7	80,5 ± 18,0	80,4 ± 17,3	0,933
BMI	27,7 ± 4,5	27,4 ± 4,6	27,5 ± 4,6	0,533
ASA-Score	2,0 ± 0,6	2,0 ± 0,6	2,0 ± 0,6	0,779
Female: Male	76:74	80:70	156:144	0,364
Operative time (min)	72,2 ± 14,3	59,1 ± 13,3	65,7 ± 15,3	**0**,**001**
Length of stay at hospital (d)	7,0 ± 2,8	6,4 ± 2,6	6,7 ± 2,7	0,055

Overall, 42 AE occurred within both study groups with a total of 27 AE (18.0%) in Group A and a total of 15 AE (10.0%) in Group B (OR = 1.98, 95% CI: 1.00–3.89; *p* = 0.046; Table 2). The sub-analysis revealed non serious AE in 17 cases (11.3%) within Group A and in 9 cases (6.0%) within Group B (OR = 2.00, 95% CI: 0.863–4.65; *p =* 0.101). Postoperative anemia and wound healing deficits were documented more often within Group A without statistical significance. In Group A one case of sintering of the femoral shaft of 3 mm occurred without any clinical symptoms or signs of loosening, which was treated conservatively. Additionally, one case of temporary abductor muscle weakness occurred, which was also treated successfully conservatively. In Group B three cases of slight postoperative cup tilting of less than 5° without any clinical symptoms or signs of loosening within the further follow-up were documented.

**Table 2:** Adverse events within the study population

	Group A (*n* = 150)	Group B (*n* = 150)	Overall (*n* = 300)	*p* Value
Overall AE	18.0% (*n* = 27)	10.0% (*n* = 15)	14.0% (*n* = 42)	**0.046**
AE	11.3% (*n* = 17)	6.0% (*n* = 9)	8.7% (*n* = 26)	0.101
Postoperative Anemia	8.0% (*n* = 12)	3.3% (*n* = 5)	5.7% (*n* = 17)	0.080
Wound healing deficit	2.0% (*n* = 3)	0.7% (*n* = 1)	1.3% (*n* = 4)	0.314
Others	1.3% (*n* = 2)	2.0% (*n* = 3)	1.7% (*n* = 5)	0.652
Serious AE	6.7% (*n* = 10)	4.0% (*n* = 6)	5.3% (*n* = 16)	0.304
PJI	2.7% (*n* = 4)	0.7% (*n* = 1)	1.7% (*n* = 5)	0.176
Periprosthetic Fracture	4.0% (*n* = 6)	2.0% (*n* = 3)	3.0% (*n* = 9)	0.310
Others	-	1.3% (*n* = 2)	0.7% (*n* = 2)	0.156
Revision	4.7% (*n* = 7)	1.3% (*n* = 2)	3% (*n* = 9)	0.091

Serious AE were documented in 10 cases (6.7%) within Group A and in 6 cases (4.0%) within Group B (OR = 1.71, 95% KI: 0.61–4.84; *p* = 0.304). Periprosthetic joint infections (PJI) were observed in 4 cases (2.7%) within Group A and in 1 case (0.7%) within Group B (OR = 4.08, 95% KI: 0.45–36.96; *p* = 0.176). Debridement, Antibiotics and implant retention (DAIR) was performed successfully in all cases of PJI. Periprosthetic fractures were observed in 6 cases (4.0%) within Group A [3 cases of postoperative Vancouver Type B2 fractures required surgical intervention (revision stem and cerclages) and 3 cases (2 cases of postoperative undisplaced Vancouver Type B1 fractures and 1 case of intraoperative undisplaced fissural acetabulum fracture without cup loosening) were treated conservatively with 4 weeks of no weightbearing followed by 2 weeks of partial weightbearing of 10 kg bodyweight] and in 3 cases (2.0%) within Group B (1 case of intraoperative Vancouver Type B2 shaft fracture required the usage of a revision stem with cerclages, 2 cases of intraoperative Vancouver Type A_G_ fractures were treated conservatively with partial weightbearing for 6 weeks; OR = 2.04, 95% KI: 0.50–8.32; *p* 0.310). In Group B two severe indirectly surgery related AE occurred (1 case of pulmonary embolism and 1 case of perforated gastric ulceration, which were both treated successfully by the medical department of the study center).

Overall, the documented AE were distributed across the study groups without statistically significant differences between early and late cases of each group (Group A: Case 1–50: 7 AE, case 51–100: 8 AE, case 101–150: 12 AE; *p* = 0.387; Group B: Case 1–50: 5 AE, case 51–100: 4 AE, case 101–150: 6 AE; *p* = 0.801).

Revisions were performed in 7 cases (4.7%; 3 cases of periprosthetic fractures and 4 cases of PJI) within Group A and in 2 cases (1.3%; 1 case of periprosthetic fracture and 1 PJI) within Group B (OR = 3.62, 95% KI: 0.74–17.73; *p* = 0.091).

**Fig. 1:**
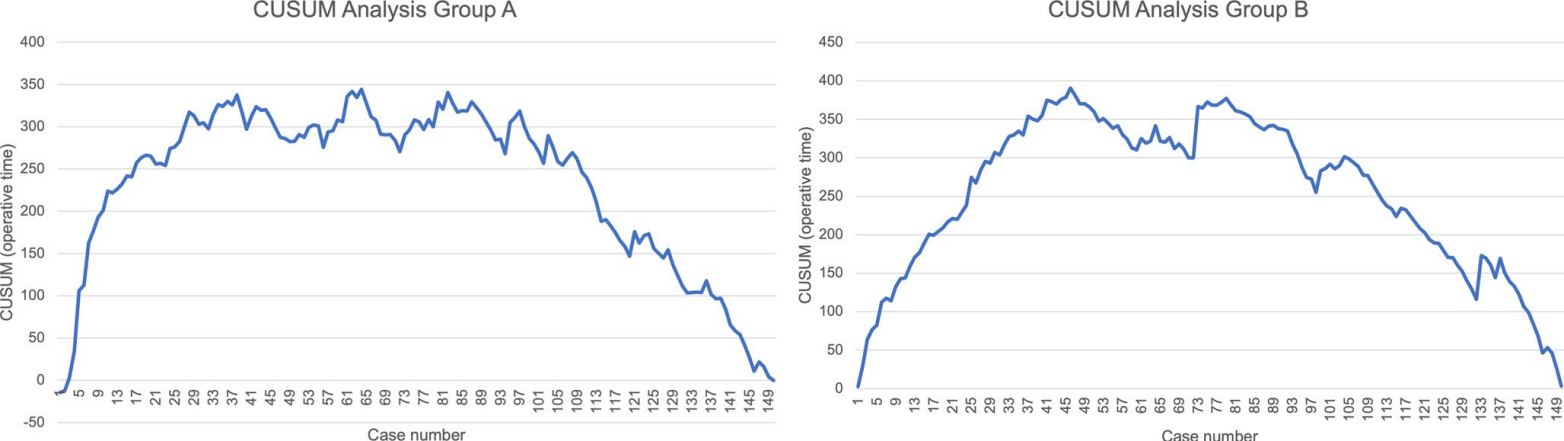
**(a)** Results of the CUSUM Analysis of the operative time of Group A. **(b)** Results of the CUSUM Analysis of the operative time of Group B

The CUSUM analysis showed a consistent decrease in operative time after the 97th case in Group A (Fig. 1a) and after the 79th case in Group B (Fig. 1b).

## Discussion

The results of this study revealed significantly more AE when switching the approach to a DAA for performing THA despite using a familiar implant compared to switching from a familiar implant to a new implant but performing THA through a familiar approach to the hip.

In general, a steep learning curve of the DAA is reported by many authors [12]. The results of this study confirm these reports by showing a considerable overall rate of AE in Group A of 18.0% and a rate of serious AE in Group A of 6.7% within the first 150 cases of performing a DAA. While other authors report significantly higher rates of AE in early DAA cases compared to late DAA cases, AE were documented without statistically significant differences across early and late cases of Group A with slightly more AE within case 101–150, which is in contrast to reports in the existing literature [11, 13]. Additionally, the overall revision rate of 4.7% within the first 150 cases of performing DAA within the first 90 postoperative days is slightly higher compared to other reports [8, 13]. The analysis of the first 150 cases of switching to a new implant while still performing a DAA (Group B) showed a significantly lower rate of overall AE of 10.0% as well as a lower rate of serious AE of 4.0%. While specific data regarding the learning curve of switching from a neck-resecting to a partially neck-sparing cementless short stem is rare compared to data regarding the DAA learning curve, there seems to be a relevant learning curve in general when switching implants for performing THA [24, 33–35].

The most frequent AE within the study population was postoperative anemia followed by wound healing deficits. As for severe AE causing revision, periprosthetic fractures were the main reason for revision followed by PJI. We observed more periprosthetic fractures in Group A compared to Group B (4.0% vs. 2.0%; *p* = 0.310), which might reflect the previously described increased risk of femoral fractures when switching to DAA [36]. While periprosthetic fractures and PJIs as main reasons for revision are also reported by other authors, there were no cases of dislocation or nerval lesions within this study population, which is in contrast to other reports, as especially lesions of the lateral femoral cutaneous nerve seem to be frequently associated with DAA [37, 38]. One case of femoral stem sintering of 3 mm in Group A and three cases of postoperative cup tilting of less than 5° in Group B with no clinical signs of aseptic loosening and no progression within the first postoperative year and therefore not causing revision were observed which is also not quite matching available data in the literature as some authors report aseptic stem loosening as a major reason for revision [13]. The three cases of cup tilting were observed in early cases (case 13, 23 and 34) within Group B with no further cases of cup tilting after further detailed instruction of the manufacturer regarding the correct milling technique for the specific cup (slight wobbling of the last fraise while maintaining a fixed rotation center before cup implantation), which might indicate a learning curve of the specific investigated cup. However, due to the study design, we are not able to determine clear reasons for the divergences of this study’s results to other reports in the literature. What has to be kept in mind though, is the wide variety of different THA settings and details like the approach performed before switching to a DAA, use of traction table or intraoperative fluoroscopy and many more factors which potentially affect the overall outcomes and types as well as rates of AE after THA.

The overall average operative time was 65.7 min (72.2 min in Group A and 59.1 min in Group B), which is relatively low compared to other reports, which state an average THA operative time above 90 min [39]. One possible reason for that comparatively low operative time is the single surgeon design of this study, which only investigated operative times of a single experienced and fellowship trained surgeon, as the inclusion of operative times of less experienced hip surgeons or trainees might raise the average operative time [40].

The CUSUM analysis of the operative times revealed a significant and consistent decrease after the 97th case when switching from an ALA to a DAA (Group A) and after the 79th case when switching implant (Group B). These results are in line with other reports stating a plateau in operative time after roughly 100 cases when switching to a DAA [11]. The steeper learning curve regarding the operative time when switching implant is one potential reason for the differences in average operative time between the two study groups. Another potential reason for the shorter operative times in Group B might be the need of a less extensively capsular release when using a partially neck-sparing short stem in combination with a DAA as the more upright femoral neck resection level required for a partially neck-sparing short stem possibly facilitates stem preparation.

There a several limitations that have to be kept in mind when interpreting the results of this study. Firstly, the retrospective study design limits the value of this study as no causal conclusion can be drawn out of the results of this study. Secondly, no randomization was performed within this study, which potentially can cause a selection bias, as possibly certain patients with specific characteristics (e.g. severe adiposity or osteoporosis) might have received other implants like for example straight stems. Furthermore, we investigated a single fellowship trained surgeon who performs approximately 130 primary knee and hip arthroplasties per year, of which roughly 70 are primary THA, with a specific setup for performing a DAA without the use of a traction table. Therefore, the results of this study might not be applicable for other setups or surgeons of different levels of experience. Lastly, due to the lack of patient reported outcomes measures and long term follow ups, we are not able to comment on a possible relevance of this study’s findings regarding the long-term clinical outcomes.

## Conclusion

These study’s findings reveal a steeper learning curve of switching implant compared to switching approach while performing THA with significantly less overall AE and an earlier decrease in operative time. However, according to the results of this study, surgeons should be aware of the learning curve associated with the switch to a new implant with different fixation philosophy as well.

## Electronic supplementary material

Below is the link to the electronic supplementary material.

Supplementary Material 1
